# Assessing the binding properties of the anti-PD-1 antibody landscape using label-free biosensors

**DOI:** 10.1371/journal.pone.0229206

**Published:** 2020-03-05

**Authors:** Michael E. Brown, Daniel Bedinger, Asparouh Lilov, Palaniswami Rathanaswami, Maximiliano Vásquez, Stéphanie Durand, Ian Wallace-Moyer, Lihui Zhong, Juergen H. Nett, Irina Burnina, Isabelle Caffry, Heather Lynaugh, Melanie Sinclair, Tingwan Sun, John Bukowski, Yingda Xu, Yasmina Noubia Abdiche

**Affiliations:** 1 Department of Protein Analytics, Adimab, Lebanon, NH, United States of America; 2 Carterra, Salt Lake City, UT, United States of America; 3 Department of Therapeutic Discovery, Amgen Research, Amgen Inc., Burnaby, BC, Canada; 4 Department of Computational Biology, Adimab, Palo Alto, CA, United States of America; 5 Department of High Throughput Expression, Adimab, Lebanon, NH, United States of America; 6 Department of Antibody Discovery, Adimab, Lebanon, NH, United States of America; New York State Department of Health, UNITED STATES

## Abstract

Here we describe an industry-wide collaboration aimed at assessing the binding properties of a comprehensive panel of monoclonal antibodies (mAbs) against programmed cell death protein 1 (PD-1), an important checkpoint protein in cancer immunotherapy and validated therapeutic target, with well over thirty unique mAbs either in clinical development or market-approved in the United States, the European Union or China. The binding kinetics of the PD-1/mAb interactions were measured by surface plasmon resonance (SPR) using a Carterra LSA instrument and the results were compared to data collected on a Biacore 8K. The effect of chip type on the SPR-derived binding rate constants and affinities were explored and the results compared with solution affinities from Meso Scale Discovery (MSD) and Kinetic Exclusion Assay (KinExA) experiments. When using flat chip types, the LSA and 8K platforms yielded near-identical kinetic rate and affinity constants that matched solution phase values more closely than those produced on 3D-hydrogels. Of the anti-PD-1 mAbs tested, which included a portion of those known to be in clinical development or approved, the affinities spanned from single digit picomolar to nearly 425 nM, challenging the dynamic range of our methods. The LSA instrument was also used to perform epitope binning and ligand competition studies which revealed over ten unique competitive binding profiles within this group of mAbs.

## Introduction

Therapeutic monoclonal antibodies (mAbs) are providing transformative medicines in treating cancer and many other life-threatening diseases, including autoimmune, heart and infectious diseases.[1, 2] The number of mAbs achieving first-market approval in the European Union or United States continues to rise annually, with 2018 delivering twelve new entities to the market and a robust clinical pipeline comprising over 570 mAbs, excluding biosimilars, of which more than 60 are in late-stage clinical evaluation.[3] For any given target there are often several pharmaceutical companies competing for fast track, breakthrough therapy, accelerated approval, or priority review, making it imperative that a new drug offers a significant benefit in this crowded commercial space. Even with these accelerated timelines, drug discovery is still a non-prescriptive and tedious process, often taking over a decade to advance a drug from the bench to the market. The high cost involved in discovering medicines compounded by the frequent failure of many programs along the way generates demand for more efficient screening and characterization methods to streamline research and cut costs when triaging from library to leads.

Label-free biosensors, such as those employing surface plasmon resonance (SPR) detection, are commonly used to guide the lead optimization process by characterizing the binding interactions of antibodies with their specific target antigens in terms of kinetic rate constants, affinities and epitope diversity with each parameter providing valuable insights toward the ultimate goal of understanding a drug’s mechanism of action. At the outset of this project our aims were threefold: 1) compare high and low throughput kinetic and affinity measurement techniques on a validated set of therapeutically relevant antibodies, 2) determine which sensor chip type (flat or 3D-hydrogel) provides affinities that more closely resemble those determined from established solution-based methods and 3) characterize the binding properties of an important class of antibodies.

We show in these studies that the high-throughput Carterra LSA (to be referred as the LSA from here on) platform provides data that compares favorably with data collected on the lower throughput Biacore 8K (referred to as 8K from here on) platform. By testing a variety of sensor chip types (flat and 3-D hydrogel) from different suppliers and across both platforms, we show that affinities measured on flat sensor chips more closely resemble data obtained from solution-based affinity techniques. This contrasts with the prevailing consensus that 3-D hydrogel sensor chips are the better choice when attempting to approximate a solution-like environment for SPR studies. Lastly, we categorized the anti-PD-1 antibody landscape in terms of kinetics of binding, diversity of epitope and ability to block the interaction between PD-1 and programmed death-ligand 1 (PD-L1). Here we were able to benchmark the binding properties of antibodies such as nivolumab and pembrolizumab, currently the standard of care for various cancer indications, with recently approved competitor antibodies and alongside antibodies still in development or of historical significance.

## Results

### Selection of a diverse anti-PD-1 mAb panel

In previous benchmarking studies, highly validated sets of antibodies derived from published sequences have been used to generate data associated with their biophysical and chemical stability properties.[4–9] We chose to do something similar for this SPR-focused study, and selected antibodies from the patent literature that target PD-1, an important checkpoint protein in immuno-oncology that helps the immune system to regulate and eliminate tumors. The effort led us to 35 mAbs that we produced as isotype IgG4, being the most common format in fully documented cases. As in past work of this kind, we opted to create isotype-matched samples, and made no attempt to reproduce the full sequences, let alone expression systems and purification processes, of the clinical molecules, in particular.[4] We should thus consider these samples as analogs of the corresponding antibody drugs or reagents.

The source of sequence information for each anti-PD-1 mAb is listed in S1 Table. We will refer to the samples in the text and figures primarily by their mAb codes and whenever an International Nonproprietary Name (INN) or its abbreviation is mentioned, it should be understood we mean the analogue sample made as mentioned above and described in more detail in the materials and methods section.

Based on the literature, we expected these antibodies to exhibit a broad range of binding affinities and epitope specificities, thereby providing reagents that would challenge our biosensor methods. The panel included ten mAbs with an assigned INN, at various stages of clinical development of which five, camrelizumab (camre), cemiplimab (cemip), pembrolizumab (pembro), nivolumab (nivo), and sintilimab (sinti), are market approved. We excluded pidilizumab from these studies, since it was recently shown not to bind to PD-1.[10] The remaining 25 mAbs span a wider range of use and include mAbs that may have advanced to various stages of clinical development and others either still in preclinical development or likely serving a control or reagent purpose; some may have historical interest. For example, we included antibodies like mAb01 and mAb14 that were part of the discovery efforts leading to nivolumab and cemiplimab, respectively, and to our knowledge were not advanced to clinical development. Others like mAb11 and mAb35 were the fruit of very early discovery efforts undertaken just around the time the important role of PD-1 as immune regulator was being elucidated.

### SPR studies show that the anti-PD-1 mAbs bind PD-1 with a broad affinity range

**Fig 1:**
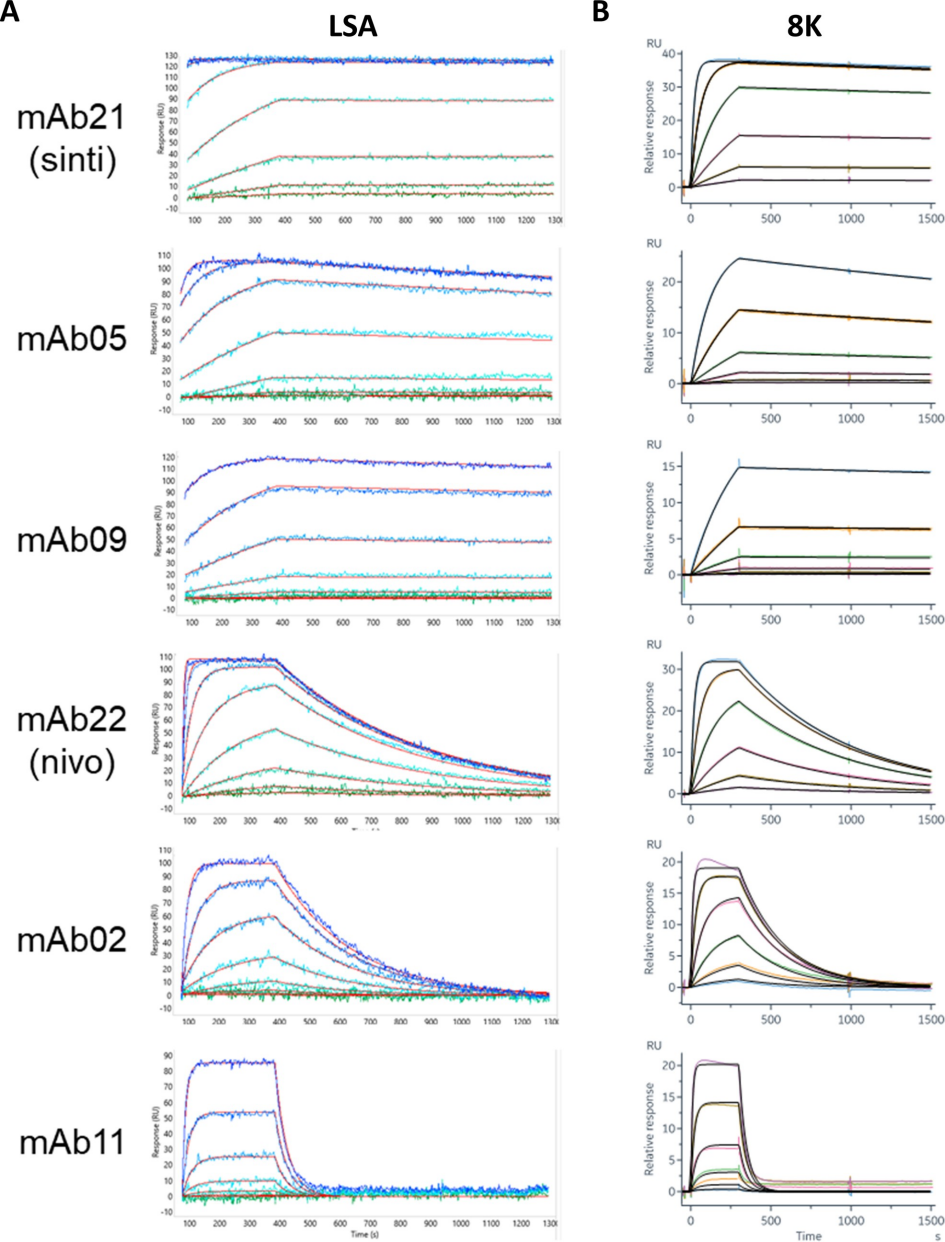
SPR capture kinetics on a CMD-P (flat) chip. (A) Examples of data from six individual spots on the LSA, representing mAbs with diverse binding kinetics. Each panel shows an overlay plot of the measured responses, as a color gradient, from green to blue, representing ascending analyte (PD-1) concentrations from 0.5 nM– 1000 nM with the global fit in red. (B) Corresponding sensorgrams collected with the Biacore 8K with analyte concentrations from 0.41 nM– 100 nM (color gradient) for mAb05, mAb09, mAb21 and mAb22; 4.1 nM– 1000 nM (color gradient) for mAb02 and mAb11. Global fits are shown in black.

To facilitate a direct comparison between the LSA and 8K instruments we designed anti-human Fc capture assays for each system. For the LSA instrument we captured each mAb onto multiple discrete spots at varying capacities to fill out a 384-ligand array. This enabled a parallel comparison of the kinetic rate and affinity constants for the interactions between the anti-PD-1 mAbs and PD-1. Fig 1A shows an overlay plot of the sensorgrams and fits obtained using the LSA instrument for six mAbs that represent the diversity of binding responses observed from these studies. By incorporating replicate ligand measurements into the array (S1 Fig) the binding kinetics could be reported with statistical confidence (Table 1). Mean apparent affinities (*K*_D_ values) spanned a > 4,000-fold range from < 100 pM to 424 nM.

**Table 1:** Mean kinetic rate and affinity constants determined from SPR experiments on the LSA instrument equipped with a flat sensor chip (CMD-P). MAbs are affinity-ranked, with upper limits reported for *k*_d_-limited mAbs (in bold)–see Materials and Methods. All data were collected in parallel on a single array, except for mAb01 and mAb35, which were analyzed in a separate experiment. Binding to PD-1 was not observed for mAb24 during this portion of the study, thus no data is reported for this interaction.

mAb ID	Analog of indicated INN	N	Mean *k*_a_ (M^-1^s^-1^) x10^5^	*k*_a_ Std. Dev. x10^5^	Mean *k*_d_ (s^-1^) x10^-4^	*k*_d_ Std. Dev. x10^-4^	Mean *K*_D_ (nM)	*K*_D_ Std. Dev.
**mAb03**	**tislelizumab**	**12**	**4.3**	**0.4**	**<0.43**	**0**	**<0.099**	**0.008**
**mAb20**		**12**	**3.4**	**0.2**	**<0.43**	**0**	**<0.127**	**0.009**
**mAb21**	**sintilimab**	**12**	**3.3**	**0.2**	**<0.43**	**0**	**<0.13**	**0.01**
**mAb19**	**sasanlimab**	**12**	**2.5**	**0.3**	**<0.43**	**0**	**<0.17**	**0.02**
**mAb18**		**12**	**2.2**	**0.1**	**<0.43**	**0**	**<0.19**	**0.01**
**mAb28**		**8**	**2.1**	**0.1**	**<0.43**	**0**	**<0.20**	**0.01**
mAb06		12	4.3	0.4	1.1	0.3	0.26	0.07
**mAb34**		**12**	**0.7**	**0.1**	**<0.43**	**0**	**<0.59**	**0.05**
mAb04	retifanlimab	12	5.8	0.5	3.9	0.6	0.7	0.1
mAb23	cemiplimab	12	1.9	0.2	2.8	0.4	1.5	0.3
mAb16	dostarlimab	12	2.4	0.1	3.9	0.2	1.6	0.1
mAb31		8	2.6	0.1	4.1	0.3	1.6	0.1
mAb32		8	7.5	0.3	14	1.2	1.9	0.2
mAb01		2	1.4	0.1	2.9	0.2	2.1	0.2
mAb27		8	6.8	0.3	17	2	2.4	0.3
mAb26		8	7.4	0.3	18	1	2.5	0.2
mAb05		12	0.5	0.0	1.7	0.3	3.1	0.5
**mAb09**		**12**	**0.13**	**0.01**	**<0.43**	**0**	**<3.2**	**0.2**
mAb29		8	1.5	0.1	4.8	0.2	3.2	0.2
mAb13	pembrolizumab	12	6.7	0.8	27	2	3.9	0.5
mAb17	camrelizumab	12	1.8	0.1	7.3	0.6	4.0	0.4
mAb33		4	3.2	0.4	17	3	6	1
mab22	nivolumab	12	3.1	0.3	22	2	7.2	0.8
mAb12		12	2.0	0.2	18	1	9	1
mAb10		12	0.9	0.1	8.7	0.5	9.4	0.8
mAb08		12	1.2	0.1	15	2	12	2
mAb15	balstilimab	12	2.4	0.1	29	2	12	1
mAb07		12	0.50	0.03	26	1	52	4
mAb02		12	0.57	0.02	38	2	67	4
mAb35		8	4.8	0.2	460	20	96	6
mAb14		12	0.29	0.01	40	4	136	14
mAb11		12	0.8	0.1	231	24	308	44
mAb25		8	1.6	0.2	691	102	424	89

The results from the LSA studies helped guide experiments with the 8K instrument. We performed similar capture experiments on the 34 highest affinity anti-PD-1 mAbs from the LSA studies and measured them in groups of eight. Fig 1B shows representative sensorgrams from this data and provides a direct and favorable qualitative comparison to the data collected with the LSA. As shown by the overlay of fits to the experimental data, data from both instruments fit well to a 1:1 Langmuir binding model.

### 3D-hydrogels produce systematically slower on-rates and therefore weaker apparent affinities than flat sensor chip surfaces

**Fig 2:**
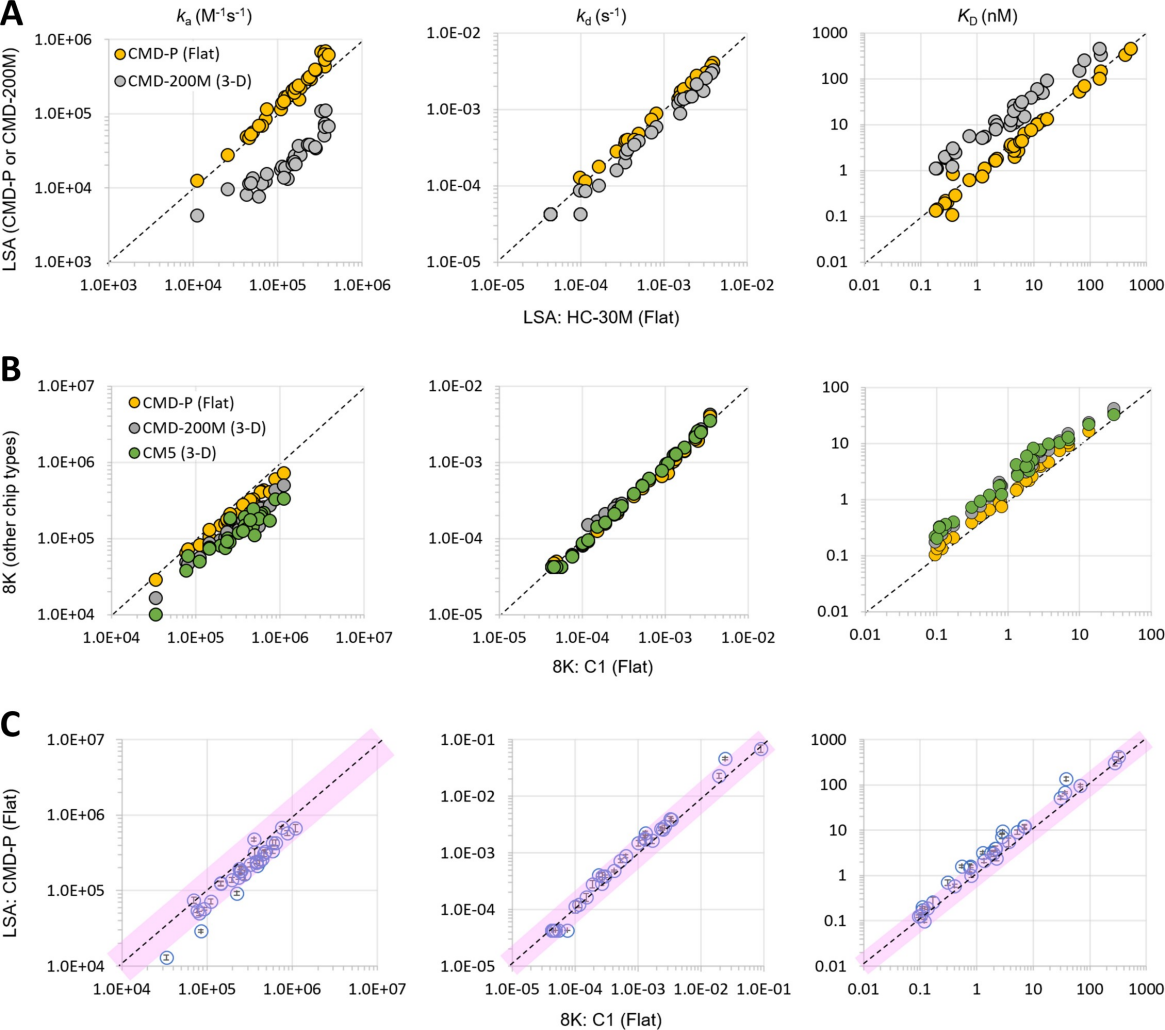
Exploring the effect of chip type on the apparent kinetic rate and affinity constants produced by the LSA and 8K instruments. (A) Scatter plots reporting the mean *k*_a_, *k*_d_, and *K*_D_ values for LSA data produced on different chip types, HC-30M (X axis) compared with CMD-P (orange) and CMD-200M (grey). The mean values were calculated from 8–12 measurements (spots) per mAb. (B) Scatter plots reporting single measurements of *k*_a_, *k*_d_, and *K*_D_ for Biacore 8K data produced on different chip types, C1 (X axis) compared with CMD-P (orange), CM5 (green) and CMD-200M (grey). (C) Benchmarking LSA-CMD-P data against Biacore 8K-C1 data, where the LSA data represent the mean (symbol) ± stdev (Y-axis error bars) for 8–12 replicate measurements (spots) per mAb. In each plot, a perfect correlation is indicated by the dashed diagonal line. The pink shaded areas define a two-fold deviation from perfect correlation. Panels A and C show data for 33 mAbs, whereas panel B shows data for 29 mAbs. Extreme outliers or data with poor fits to a 1:1 biding model were excluded.

To determine the effect of chip type on the apparent kinetic rate constants, we performed the capture kinetics experiments using both flat chips and 3D-hydrogels. The results from the LSA instrument showed that 3D-hydrogels (CMD-200M) systematically produced 3- to 14-fold weaker affinities (higher *K*_D_ values) than flat chips (CMD-P and HC-30M), driven almost entirely by slower on-rates (smaller *k*_a_ values) with minimal to no influence on the off-rates (*k*_d_ values) (Fig 2A). We performed similar experiments (CMD-200M and CMD-P) on the 8K instrument and were able to expand the types of sensor chips to include additional flat (C1) and 3D-hydrogel (CM5) sensor chips. Consistent with the LSA results, experiments on the 8K also showed that 3D-hydrogels produced systematically slower on-rates and weaker affinities than flat chips, with no discernible effect on the off-rate (Fig 2B). However, the perturbation was more moderate on the 8K than the LSA, with 3D-hydrogels producing on average a twofold slower on-rate (and concomitant average twofold weaker affinity) than flat chips. These results also showed very little difference between like chip types (flat v 3-D hydrogel) from different suppliers. As shown in Fig 2B, rate constants and affinities determined from C1 (GE) sensor chips correlate well with results from a CMD-P (Xantec) chip. Likewise, rate constants and affinities measured using a CM5 (GE) chip overlaid consistently with those measured on a CMD-200M (Xantec) sensor chip. Fig 2C shows that when we benchmark LSA data against 8K data using flat sensor chips (CMD-P for LSA and C1 on the 8K) we obtained remarkably similar results, yielding *k*_a_ and *k*_d_ values that were generally within twofold across platforms for a panel of 33 mAbs representing a broad range of affinities.

To assess inter-assay reproducibility, we repeated experiments on both SPR biosensor platforms. For the 8K, we duplicated the analysis for 8 mAbs using a C1 sensor chip. Given the LSA instrument’s expanded ligand capture capacity, we retested the entire mAb panel, incorporating multiple replicates per assay for two different chip types, CMD-P and HC-30M. Both biosensor platforms yielded excellent inter-assay reproducibility with near linear trend lines (S2 Fig).

Sensorgrams with fit overlays from each Biacore experiment are provided in the associated S1 File.

### Affinities produced on flat chips more closely resemble solution phase measurements

**Fig 3:**
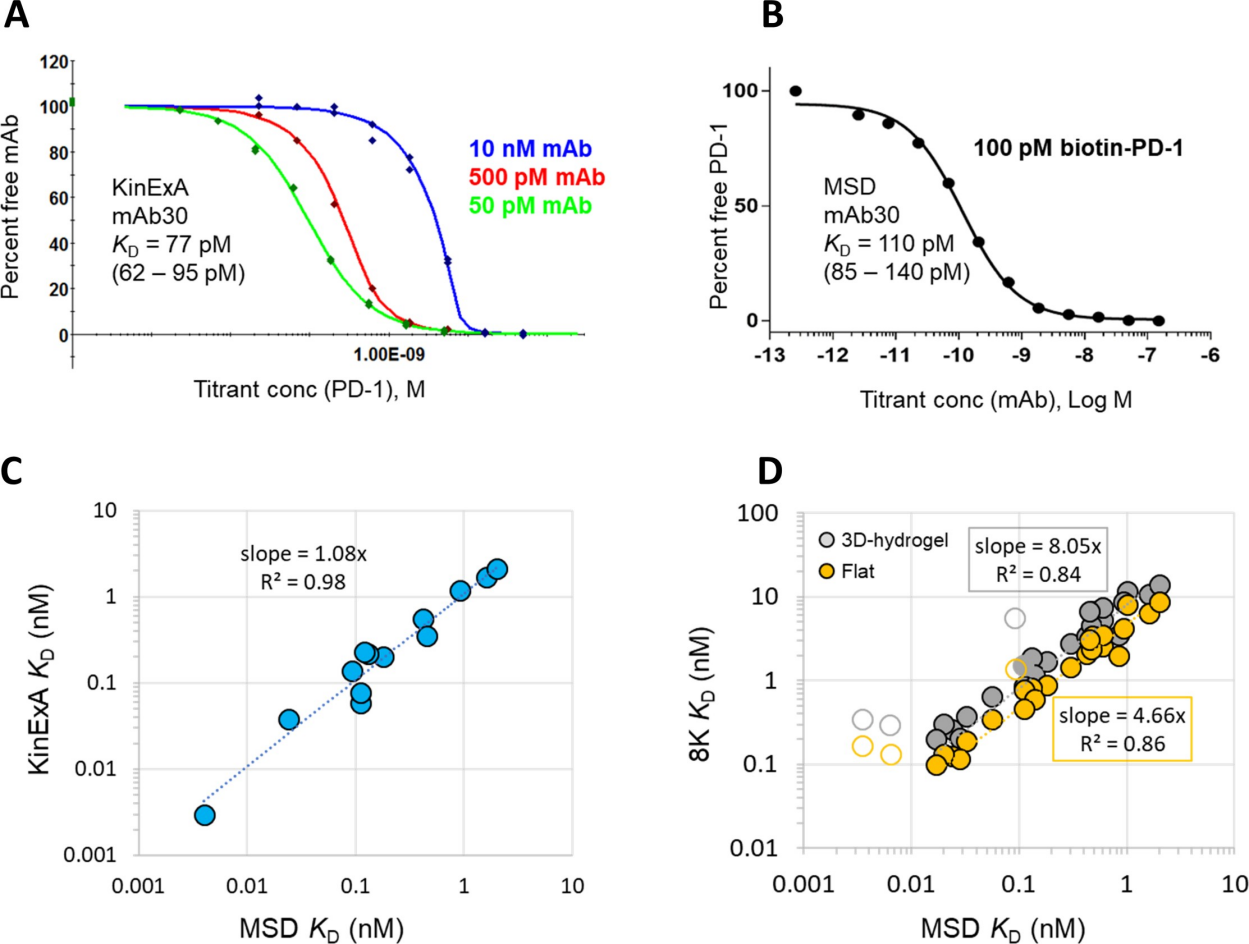
Solution affinities via KinExA and MSD. Representative solution affinity curves for mAb30 via (A) KinExA (n-curve analysis) and (B) MSD (single curve analysis). (C) Correlation between KinExA (Y-axis) and MSD (X-axis) for 13 mAbs. (D) Correlation between SPR (Y-axis, average *K*_D_ for Biacore 8K) for 27 mAbs on 3D-hydrogel (CM5 and CMD200 chips, grey) or flat chip (C1 and CMD-P, orange) and MSD (X-axis). The slope of the trendlines indicate average deviation from a perfect correlation for each chip type. Data for antibodies (mAb09, mAb18 and mAb29) measured at the off-rate limit of detection (< 4.27 x 10^−5^ s^-1^) for the SPR experiments where the correlation does not conform are indicated with open circles, and not included in the trendline calculation. Data from mAb27 and mAb33 were excluded from this plot due to poor fits to a 1:1 binding model (see the associated S1 File).

To determine which chip type (flat or 3D-hydrogel) produced affinities that more closely recapitulated solution phase measurements, we determined solution affinities using KinExA and MSD. KinExA experiments were performed on 14 mAbs, and Fig 3A shows a representative example of an N-curve solution affinity determination using the KinExA method for mAb30. We studied 30 mAbs via the MSD method, and Fig 3B shows a representative single curve solution affinity determination for the same mAb30 as well.

Both solution-based methods confirmed that this set of anti-PD-1 mAbs bind to PD-1 with a wide range (nearly 1500-fold) of affinities (*K*_D_ values ranging from 3.5 pM to 5.2 nM). Consistent with the literature,[11] we found excellent agreement between the affinities determined by KinExA and MSD (Fig 3C), and therefore used the MSD-derived solution affinities for comparisons to the SPR-derived apparent affinities. For the purpose of comparing chip type, we chose the SPR platform on which we had explored the most chip types, which was the 8K instrument, owing to the availability of Biacore’s own chips and Xantec surfaces. The results obtained on flat chips (C1 and CMD-P) were averaged and compared to the averaged values from 3D-hydrogels (CM5 and CMD-200M). Excluding data for off-rate limited mAbs, as they break the correlation, the plot in Fig 3D shows that affinities determined from flat sensor chips were on average within fivefold of those measured by MSD, whereas affinities from 3-D hydrogel surfaces were nearly within eightfold (on average) of the solution-based affinity measurements. These data are summarized in Table 2 and show a general trend in which *K*_D_ values determined by MSD < SPR (flat chip) < SPR (3D-hydrogel chip).

**Table 2:** Direct comparison of the apparent affinities (*K*_D_ values, nM) determined by solution methods (MSD and KinExA) and SPR (Biacore 8K) on flat chips (average of C1 and CMD-P) and 3D-hydrogels (average of CM5 and CMD200M). Thirty-two mAbs are included for this comparison and are ranked by their MSD/SPR affinity correlation (best to worst). ND = not determined; NB = non-binder in the assay; Bold = *k*_d_-limited in the Biacore SPR capture-based assay. See Fig 4B.

mAbID	Analog of indicated INN	MSD *K*_D_ (nM)	KinExA *K*_D_ (nM)	Biacore *K*_D_ (nM) flat	Biacore *K*_D_ (nM) 3D-hydrogel	*K*_D_ (flat)/ MSD ratio	*K*_D_ (3D-hydrogel) /MSD ratio
mAb29		0.84	ND	2.0	3.5	2	4
mAb12		1.60	1.69	6.3	11	4	7
**mAb21**	**sintilimab**	**0.028**	**ND**	**<0.12**	**<0.21**	**4**	**7**
**mAb34**		**0.11**	**0.059**	**<0.46**	**<0.85**	**4**	**8**
mAb31		0.14	ND	0.60	1.2	4	8
mAb13	pembrolizumab	0.59	ND	2.5	5.3	4	9
mAb15	balstilimab	2.00	2.13	8.7	14	4	7
mAb33		0.92	1.2	4.2	8.8	5	10
mAb01		0.30	ND	1.5	2.8	5	9
mAb16	dostarlimab	0.18	0.2	0.87	1.7	5	9
mAb05		0.42	0.55	2.1	3.4	5	8
mAb17	camrelizumab	0.47	ND	2.4	4.5	5	10
mAb03	tislelizumab	0.024	0.038	0.13	0.26	5	11
**mAb20**		**0.017**	**ND**	**<0.10**	**<0.20**	**6**	**12**
mAb22	nivolumab	0.60	ND	3.5	7.4	6	12
mAb06		0.032	ND	0.19	0.37	6	12
mAb04	retifanlimab	0.056	ND	0.35	0.65	6	12
mAb23	cemiplimab	0.13	0.22	0.82	1.9	6	14
**mAb19**	**sasanlimab**	**0.020**	ND	**<0.13**	**<0.30**	**7**	**15**
mAb27		0.45	0.35	3.0	6.7	7	15
mAb30		0.11	0.077	0.77	1.5	7	14
mAb10		0.48	ND	3.4	6.7	7	14
mAb08		1.00	ND	8.0	12	8	12
**mAb09**		**0.092**	**0.14**	**<1.4**	**<5.5**	**15**	**60**
**mAb28**		**0.0064**	**ND**	**<0.13**	**<0.30**	**21**	**46**
mAb32		0.12	0.225	2.6	5.0	22	42
**mAb18**		**0.0035**	**0.0035**	**<0.17**	**<0.34**	**48**	**97**
mAb26		0.036	ND	2.8	5.1	79	140
mAb07		NB	ND	33	37	ND	ND
mAb24		NB	5.2	15	23	ND	ND

**Fig 4:**
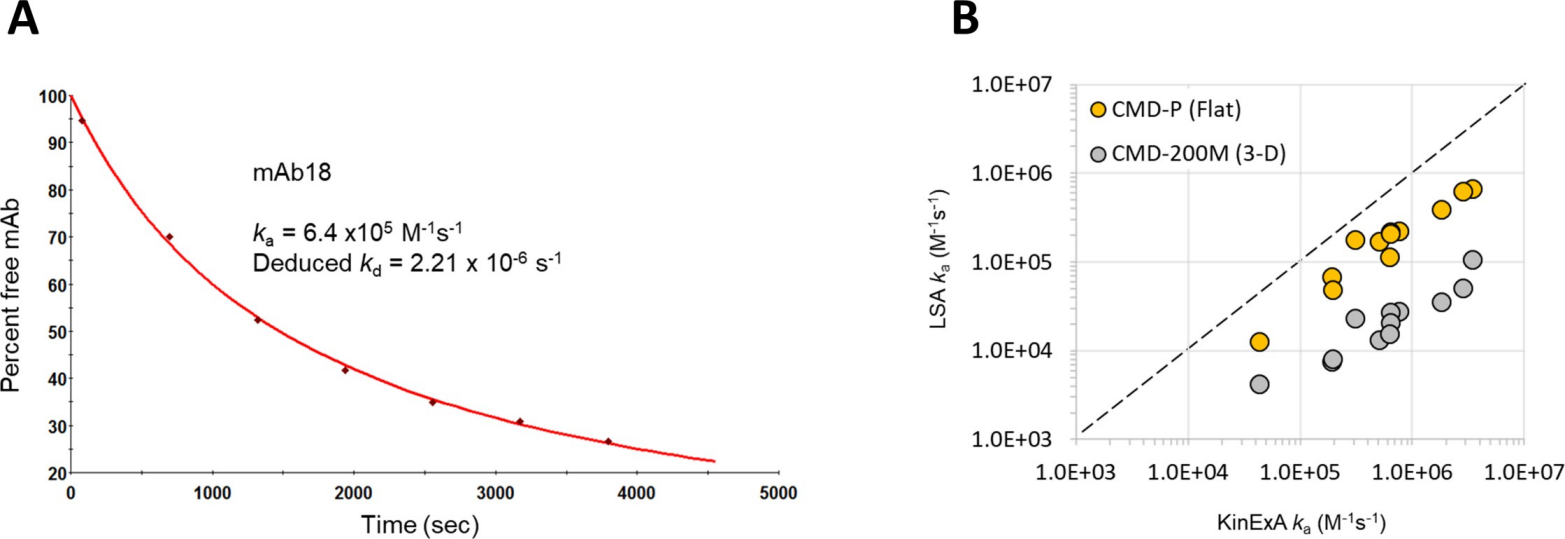
Benchmarking on-rates determined on different chip types using high-throughput SPR (LSA) versus solution phase measurements (KinExA). (A) Example result from a KinExA on-rate experiment (mAb18). (B) Correlation plot showing the effect of chip type (flat or 3D-hydrogel) on the apparent on-rates determined by the LSA (Y-axis) benchmarked against solution values measured on the KinExA (X-axis).

To further confirm which chip type (flat or 3D-hydrogel) produced on-rates that correlated more strongly with solution phase values, we performed a series of on-rate experiments with 13 mAbs using the KinExA instrument. Fig 4A shows an example of a typical “direct method” KinExA on-rate experiment. For these experiments the mAb and antigen are mixed and set on a course to reach equilibrium. By measuring free mAb in solution as a function of time one can directly measure the association rate constant.[12] Using the known affinity from equilibrium-based affinity measurements one can then deduce the dissociation rate constant from these data. Using LSA data produced on CMD-P (flat) and CMD-200M (3D-hydrogel) chip types as examples, we show (Fig 4B) that the KinExA-determined on-rates more closely resemble on-rates measured with flat chips than 3D-hydrogels. S2 Table summarizes the results from the KinExA on rate experiments and provides the input SPR data used to produce Fig 4B.

Solution affinity curves with fit overlays for the MSD experiments are provided in the associated S1 File.

### High-throughput SPR revealed exquisite epitope differences between the anti-PD-1 mAbs

**Fig 5:**
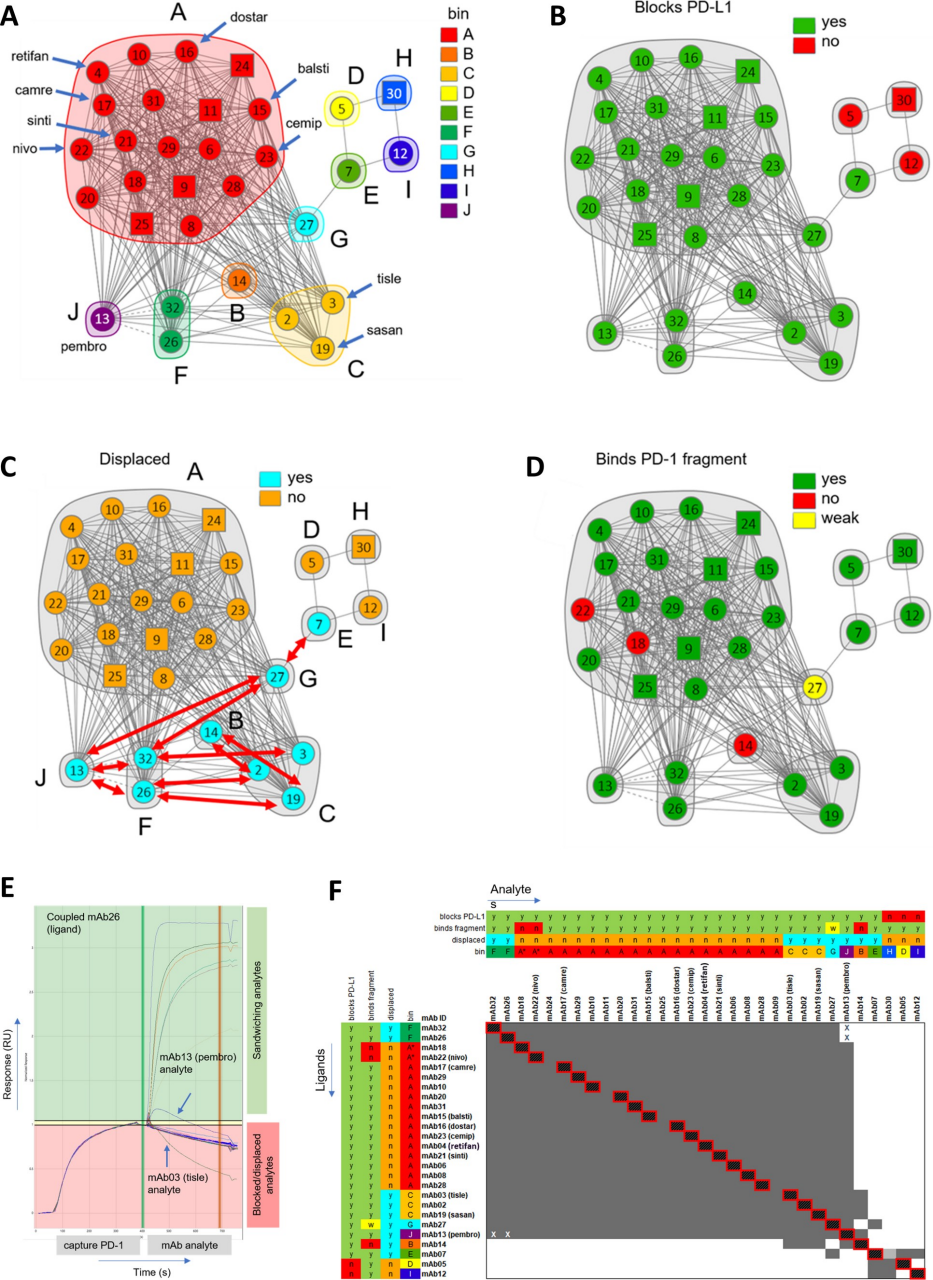
Network plots depicting the epitope clusters deduced from binning a panel of 31 anti-PD-1 mAbs. Blocking relationships (cords) between mAbs (nodes) enable the clustering of mAbs into bins (inscribed by envelopes), where a bin represents a family of mAbs sharing an identical blocking profile when tested against all other mAbs in the test set. The plots are colored by (A) bin, (B) PD-L1 blockade (green = blocks, red = does not block), (C) displacement (cyan = yes, orange = no), where the red arrows indicate the observed displacements and (D) binding to P35-Q167 PD-1 fragment (green = binds, red = does not bind, yellow = binds fragment with weaker affinity than full-length PD-1). All mAbs were tested as both analyte and ligand (circular nodes) except five mAbs (09, 11, 24, 25, and 30) which were tested only as analyte (square nodes). (E) Sensorgram overlay plot depicting an example of displacement. In this example, analytes mAb13 (pembro) and mAb03 (tisle) each displace PD-1 from coupled mAb26, as judged by sandwiching curves that show decaying or inverted responses, respectively.[14] This implies that mAb03 and mAb13 each target epitopes that are closely adjacent to or minimally overlapping with that of mAb26. (F) Heat map merged with orthogonal data where grey represents a blocking relationship and white a sandwich (non-blocking). The cells marked with an X indicate an asymmetric block, i.e., an apparent block in only one direction of the heatmap (mAb13 vs mAb26 and mAb13 vs mAb32), due to the formation of a transient sandwiching complex observed in only one order of addition, consistent with a displacement.

We explored the epitope coverage of these anti PD-1 mAbs by performing pairwise competition or "epitope binning" experiments via high-throughput SPR on the LSA instrument, as described elsewhere.[13] Fig 5 summarizes the results from these binning experiments as graphed network plots. Ten bins emerged from this study as shown in Fig 5A, where the networks are colored by bin. When combined with orthogonal data, these bins were categorized into sub-bins. For example, Fig 5B shows that mAb05, mAb12, and mAb30 were the only antibodies unable to block binding to PD-L1. This figure also shows that mAb07, a PD-L1 blocker, represents mAbs that bind to a bridging epitope between PD-L1-blocking and non-blocking antibodies. Interestingly, since the majority of mAbs targeted subtly different ligand-blocking epitopes, several mAbs displaced one another (Fig 5C and 5E), implying that these mAbs targeted closely adjacent or minimally overlapping epitopes.[14] These data are summarized in Fig 5F and Table 3.

**Table 3:** Summary of the epitope coverage and binding affinities of 31 anti-PD-1 mAbs. (*K*_D_ values represent the mean values from 8–12 spots per mAb, as determined on the LSA using a CMD-P chip; ND = not determined; *k*_d_-limited in SPR capture-based assay in bold). Data for mAb33 and mAb34 were determined in a separate experiment. The mAbs are sorted by Bin. (see Fig 5).

mAb ID	Analog of indicated INN	LSA *K*_D_ (nM)	Bin	Blocks PD-L1	Binds P35-Q167 PD-1 fragment	Shows mAb Displacement	Displaced mAbs
mAb22	nivolumab	7.2	A*	Y	N	N	
**mAb18**		**<0.2**	A*	**Y**	**N**	**N**	
mAb04	retifanlimab	0.7	A	Y	Y	N	
mAb06		0.3	A	Y	Y	N	
mAb08		12	A	Y	Y	N	
**mAb09**		**<3.2**	**A**	**Y**	**Y**	**N**	
mAb10		9.5	A	Y	Y	N	
mAb11		308	A	Y	Y	N	
mAb15	balstilimab	12	A	Y	Y	N	
mAb16	dostarlimab	1.6	A	Y	Y	N	
mAb17	camrelizumab	4	A	Y	Y	N	
**mAb20**		**<0.1**	**A**	**Y**	**Y**	**N**	
**mAb21**	**sintilimab**	**<0.1**	**A**	**Y**	**Y**	**N**	
mAb23	cemiplimab	1.5	A	Y	Y	N	
mAb24		ND	A	Y	Y	N	
mAb25		424	A	Y	Y	N	
**mAb28**		**<0.2**	**A**	**Y**	**Y**	**N**	
mAb29		3.2	A	Y	Y	N	
mAb31		1.6	A	Y	Y	N	
mAb33		6	A	ND	ND	ND	
**mAb34**		**<0.59**	**A**	**ND**	**ND**	**ND**	
mAb14		136	B	Y	N	Y	mAb02, 19
mAb02		67	C	Y	Y	Y	mAb14, 26
**mAb03**	**tislelizumab**	**<0.1**	**C**	**Y**	**Y**	**Y**	mAb32
**mAb19**	**sasanlimab**	**<0.2**	**C**	**Y**	**Y**	**Y**	mAb14, 26
mAb05		3.1	D	N	Y	N	
mAb07		52	E	Y	Y	Y	mAb27
mAb26		2.5	F	Y	Y	Y	mAb02, 13, 19
mAb32		1.9	F	Y	Y	Y	mAb03, 13, 27
mAb27		2.4	G	Y	W^#^	Y	mAb07, 13, 32
mAb30		1	H	N	Y	N	
mAb12		9.2	I	N	Y	N	
mAb13	pembrolizumab	4	J	Y	Y	Y	mab26, 27, 32

^#^ Indicates weaker binding to N-terminal deleted PD-1.

It has been reported that the N-terminal loop of PD-1 is crucial to the binding of nivolumab.[15] We therefore prepared a construct encoding a truncated form of PD-1 (residues P35-Q167), which had the N-terminal domain deleted, and used it to screen the mAbs. In addition to mAb22 (nivo), mAb14 and mAb18 bound wild-type PD-1 but not the fragment, while mAb27 showed a 5-fold weaker affinity to the fragment when compared with full length PD-1 (Fig 5D).

## Discussion

### Selected mAbs bind PD-1 with a wide range of affinities and subtly unique binding specificities

The anti-PD-1 mAbs in this study were selected to represent the diversity in kinetics, affinity, and epitope specificity to this important therapeutic target. We included established, approved molecules as represented by the analogs of nivo (mAb22) and pembro (mAb13), along with analogs of more recently approved mAbs, camre (mAb17), cemip (mAb23) and sinti (mAb21). Other INN-registered molecules included were balstilimab (balsti, mAb15), dostarlimab (dostar, mAb16), retifanlimab (retifan, mAb04), sasanlimab (sasan, mAb19) and tislelizumab (tisle, mAb03). The rest of the panel was comprised of 25 mAbs with sequence information available from the patent literature, several of which having been reported to have unique binding properties as compared to nivo or pembro.

The kinetic rate and affinity constants (*K*_D_ values) for nivo and pembro listed with the European Medicines Agency suggest that pembro (29 pM) binds with significantly higher affinity to PD-1 than does nivo (3.06 nM).[16, 17] In contrast, a recent publication reported the affinity for pembro to human PD-1 at 3.4 nM.[18] Discrepancies like this populate the biosensor literature, and make it difficult to compare published data, especially data where sensorgrams with fits are not shown. Since samples based on these and other INN-registered mAbs are often used as positive controls, we used these studies as an opportunity to thoroughly characterize their binding properties. By running these studies in parallel, multiple times, while using well characterized and defined binding partners, we significantly increase the reliability of these measurements.

Using data collected on the LSA equipped with a flat chip (CMD-P), we determined the affinities (*K*_D_ values) of our analogs of pembro and nivo (mAb13 and mAb22, respectively) to be 3.9 ± 0.5 and 7.2 ± 0.8 nM, respectively (n = 12). These affinities are moderate by most standards for current biologics, and more importantly, nowhere near the low pM values reported elsewhere.[17, 19, 20] The reported low pM affinity for nivo appears to be a simple mistake (citing 3.06 pM when the original source reported 3.06 nM) in citing data from an EMA document.[16] The low pM affinity reported for pembro has been mentioned in numerous peer reviewed journals.[20–22] Most articles cite an early report by Hamid et al.[23] from when pembro was referred to as lambrolizumab that included the statement “The variable region sequences of a very-high-affinity mouse antihuman PD-1 antibody (dissociation constant, 28 pM) were grafted into a human IgG4 immunoglobulin…”. The authors provided no details on how the measurements were made, did not provide sensorgrams nor did they provide a reference to support the claim and thus brings into question whether avidity played a role in this well cited measurement.

We show (Table 1) that most of the INN-analog mAbs bind with similar affinities to mAb13 (pembro) and mAb22 (nivo), with the exception of mAb03 (tisle), mAb19 (sasan), and mAb21 (sinti), each of which measures at the off-rate limit of detection (4.27 x10^-5^ s^-1^) for this assay. Of the samples corresponding to the five approved anti-PD-1 molecules (mAb34 is a closely related analog of toripalimab), mAb21 (sinti) has the highest affinity (< 130 pM) to human PD-1.

By performing a comprehensive epitope binning analysis, we found that most of the mAbs in this study belonged to the same bin as nivo (Bin A). However, based on our binding data to the PD-1 fragment, a sub-bin (Bin A*) emerged comprising mAb22 (nivo) and mAb18, which, unlike other Bin A members, required presence of the N terminus of PD-1 for binding. It is known from crystal structures that pembro and nivo target partially overlapping epitopes, and that nivo requires the N-terminal loop of PD-1 whereas pembro does not.[15] Our study not only reaffirmed this, but was able to probe finer epitope differences between them owing to the use of many more mAbs in our binning study. Thus, we assigned mAb22 (nivo) to BinA* and mAb13 (pembro) to Bin J. Additionally, mAb13 (pembro) and mAbs from Bin C, which include mAb02, mAb03 (tisle), and mAb19 (sasan), were able to displace other mAbs (mAb14, mAb26, mAb27, and mAb32), suggesting that their epitopes were closely adjacent to the epitopes of mAbs in Bins C (mAb14) and F (mAb07), see Fig 5D. We found that analogs of the other INN-designated mAbs, mab04 (retifan), mAb15 (balsti), mAb16 (dostar), mAb17 (camre), mAb21 (sinti) and mAb23 (cemip), all belonged to Bin A. No Bin A (or Bin A*) mAb participated in displacements with other bins, whereas mAbs from Bins C-G and J displaced one another, consistent with these bins targeting closely adjacent or minimally overlapping epitopes relative to one another. Of all the PD-L1-blocking mAbs, mAb07 (Bin E) had the most distinct/unique epitope, as it competed with one PD-L1 blocker, mAb27, and two PD-L1 non-blocker mAbs, mAb05 and mAb12 (Fig 5F). These subtle but unique binding profiles are summarized in Table 3.

Overall, the kinetic, affinity, and epitope landscape revealed by studying this set of anti-PD-1 mAbs shows that no “one size fits all” when it comes to choosing clinically relevant leads. In fact, a recent paper reported a novel mechanism by which PD-L1 non-blocking anti-PD-1 mAbs can show favorable anti-tumor activity.[24] In another recent publication, researchers describe a set of potent macrocyclic peptide and small-molecule PD-1/PD-L1 inhibitors, one of which induced cytokine production (IL-2 and IFN-γ) and T cell proliferation at levels comparable to pembrolizumab.[25] We suspect the unique binding properties observed for many of the antibodies studied here will translate to similarly unique biological activity at the cellular and potential therapeutic level.

### Flat sensor chips provide data that correlates most favorably with solution-based measurements

A technical finding from this work was the correlation of the apparent on-rate (*k*_a_ value) with sensor chip type when using SPR to determine binding affinities. We found, irrespective of biosensor platform, that carboxymethyldextran (CMD) 3D-hydrogels produced systematically slower apparent on-rates when compared to flat chips. Data from the 8K instrument showed a twofold difference, while LSA data showed a more variable difference (3-14fold). Both platforms showed that chip type had minimal to no effect on the apparent off-rate (*k*_d_ value). The observation that 3D-hydrogels produce slower on-rates than flat chips, with insignificant perturbation in the off-rate, has been reported by other investigators on other antigen/mAb pairs in carefully controlled SPR experiments.[26, 27] These studies have also shown that flat chips more faithfully recapitulate solution phase affinities. Therefore, a practical disadvantage of using CMD 3D-hydrogels is that their under-estimation of true affinities requires using unnecessarily high antigen concentrations, resulting in higher antigen consumption. In our experience, sourcing high quality antigen preparations for use as well-behaved analytes in binding kinetic and affinity determinations is often expensive, making these reagents rather precious.

Historically, planar surfaces have been reserved for special situations such as when working with multivalent interaction partners to dilute out the avidity of bivalent analytes,[28] or where carboxymethyl dextran interferes with the interaction of interest.(28) We immobilized an anti-human Fc antibody to both planar chip surfaces and found that C1 sensor chips have a nearly fourfold lower immobilization capacity when compared to the CMD-P chip surface, suggesting that the C1 chip may have fewer functional groups than CMD-P. It was from these chip surfaces that we were able to measure affinities that more closely resembled those from solution phase experiments. Dextran-based chips such as the CM5 and CMD200 are touted to provide a flexible, more “solution-like” immobilization environment owing to the relatively free movement of attached ligands within the surface layer. Indeed, with significant assay optimization, kinetic rate and affinity constants for antigen/mAb interactions measured using 3D-hydrogels can agree within twofold of solution phase values.[29] Conversely, the results from this work indicate that ready access to active ligand, as provided using a flat chip, is a more consistent and reliable system for delivering solution-like affinities.

The observation that biosensor experiments performed on flat chip types give better correlation with solution phase values than the traditional use of 3D-hydrogels, establishes a new paradigm for choosing appropriate chip types when studying antigen/mAb binding interactions. For small molecule analysis, it has been established in the literature that affinity measurements performed on Biacore using CM5 chips can show excellent agreement with those performed in solution, such as using isothermal titration calorimetry,[30] suggesting that the diffusion properties of small molecule analytes are not perturbed by the 3D-hydrogel. Indeed, the use of 3D-hydrogels is often required to create high enough ligand surface capacities to enable the detection of small molecule analytes. In contrast, when studying the binding interaction of protein antigens (large analytes) to immobilized or captured mAbs (ligands), low capacity surfaces are preferred, obviating the need for 3D-hydrogels.

## Materials and methods

### Antigens

Recombinant purified human PD-1 protein, residues Leu25-Thr168, with a C-terminal 6-His tag was purchased from R&D Systems (catalog# 8986-PD, lot# DDJM0417041), and its molecular weight confirmed by HPLC-MS and shown to be 99% pure by SEC-HPLC. It was used as an analyte in the affinity determinations and binning experiments. Monomeric human PD-1 Fc fusion protein was expressed as a murine IgG1 fusion by pairing FLAG-murine IgG1 Fc with His-tagged human PD-1 murine IgG1 Fc. The fusion protein was purified by a two-step process (anti-FLAG; Ni-NTA), was sequence confirmed by HPLC-MS and shown to be 96% pure by SEC-HPLC. It was used as a bead-coating reagent in the KinExA experiments. Recombinant purified human PD-L1 Fc chimera protein was purchased from R&D Systems (catalog# 156-B7). It was used as a competitor in the binning assays. His-tagged (9-His) N-terminal truncated PD-1, residues Pro35-Glu167, was expressed in HEK293 suspension cells, purified in a single step (Ni-NTA) to a monomeric purity of 42% (SEC-HPLC) and shown to bind (Bio-layer Interferometry, ForteBio) to pembrolizumab (100 nM) but not to nivolumab (100 nM). It was used as an analyte for affinity determinations.

### Antibody cloning, expression, purification and characterization

Thirty-five anti-PD-1 mAbs were chosen from the patent or World Health Organization-INN literature (S1 Table) to provide a panel with diverse antigen-binding properties. All mAbs were expressed as IgG4 isotype with a stabilizing ^226^CPPC hinge modification. The VH and VL encoding gene fragments (Integrated DNA Technologies) were subcloned into heavy- and light-chain pcDNA 3.4+ vectors (Thermo Fisher Scientific). The corresponding vectors were cotransfected into HEK293 suspension cells. After 6 days of growth, the cell culture supernatant was harvested by centrifugation and passed over Protein A agarose (MabSelect SuRe; GE Healthcare Life Sciences). The bound mAbs were then washed with PBS and eluted with buffer (200 mM acetic acid/50 mM NaCl, pH 3.5) into 1/8 volume 2 M Hepes, pH 8.0. The final products were buffer exchanged into 25 mM Hepes and 150 mM sodium chloride, pH 7.3. Their purity, homogeneity, and binding activity were confirmed by various analytical methods, including molecular weight confirmation by LC-MS, SEC-HPLC (86–99% monomer), and binding to CHO-S cells expressing human-PD-1 (76–1260 fold over background to parental cells when tested at 10 nM IgG by FACS).

### Capture kinetics using the Biacore 8K

A Biacore 8K SPR system (GE HealthCare) equipped with different sensor chip types, flat (Biacore C1 and Xantec CMD-P) and 3D-hydrogel (Biacore CM5 and Xantec CMD-200M), was used to generate binding kinetic rate and affinity constants at 25°C and in a running buffer of HBS-EP+ (10 mM HEPES pH 7.4, 150 mM NaCl, 3 mM EDTA, 0.05% Tween-20). The same experimental procedure was repeated on all four chip types to determine their effect on the apparent kinetic rate constants. To prepare the capture surfaces, goat anti-human IgG Fc-specific polyclonal mAb (Southern Biotech, catalog# 2014–01) was amine-coupled under standard conditions at a flow rate of 10 μL/min, as follows. Flat chip types C1 and CMD-P were preconditioned prior to coupling, using 2x 60-sec injections of glycine pH 12; no preconditioning was performed for the 3D-hydrogel chip types CM5 and CMD-200M. Flow cells 1 and 2 were activated with a freshly prepared 1:1 v/v mixture of aqueous stocks of 0.4 M 1-ethyl-3-(3-dimethylaminopropyl) carbodiimide (EDC) + 0.1 M N-hydroxysuccinimide (NHS) for 7 min. The goat anti-human IgG Fc polyclonal mAb was diluted to 12.5 μg/mL or 25 μg/mL in 10 mM sodium acetate pH 5.0 and coupled for 7 min. Finally, excess reactive esters were blocked with 1 M ethanolamine. HCl pH 8.5 for 7 min. This protocol yielded the following coupled levels (Response Units, RU), expressed as mean ± standard deviation (stdev) for 16 surfaces, representing the total data from flow cell 1 (reference) and flow cell 2 (active) for 8 channels: 1126 ± 24 (C1), 4337 ± 531 (CM5), 4210 ± 38 (CMD-P), and 9256 ± 1169 (CMD-200M). Following a stabilization period in running buffer, the anti-PD-1 mAbs (diluted to 2 μg/mL were captured onto flow cell 2 for 30–60 sec, giving captured levels (in RU) of 64–167 (C1), 48–184 (CM5), 83–210 (CMD-P), and 50–155 (CMD-200M). Recombinant purified human PD-1 His-tagged monomer was prepared at nominal concentrations of 0, 0.4, 1.2, 3.7, 11, 33, and 100 nM and injected over flow cells 1 and 2 for 5 min at a flow rate of 30 μL/min, allowing a 20-min dissociation phase at a flow rate of 30 μL/min. A duplicate injection of the 33 nM sample was performed near the end of run to assess the assay’s reproducibility. Three separate buffer blank injections were run per interaction, to provide blanks for double-referencing the data. Samples were injected in a multi-cycle manner over freshly captured mAb, by regenerating the capture surfaces with two injections of glycine pH 1.5 (20–60 s) at a flow rate of 30 μL/min. The data was processed and analyzed with Biacore 8K Evaluation Software Version 1.0 (GE Healthcare Bio-Sciences) as follows. Responses from flow cell 1 (reference) were subtracted from the responses from flow cell 2 (active). The responses from the nearest buffer blank injection were then subtracted from the reference subtracted data (2–1) to yield double-referenced data,[31] which were fit to a simple 1:1 Langmuir binding model with mass transport to determine the apparent association (*k*_a_) and dissociation rate constants (*k*_d_). Their ratio provided the apparent equilibrium dissociation constant or affinity constant (*K*_D_ = *k*_d_/*k*_a_). MAbs showing minimal decay in their binding responses within the allowed 20-min dissociation phase, were assigned a limit of *k*_d_ < 4.25 x 10^−5^ (1/s), according to the 5% rule;[32] we refer to these mAbs as “*k*_d_-limited”.

### Capture kinetics using the Carterra LSA

High-throughput SPR capture kinetic experiments were performed on Carterra’s LSA system equipped with Xantec CMD-P (flat), HC-30M (nearly flat), and CMD-200M (3D-hydrogel) chip types. All 35 anti-PD-1 mAbs were analyzed in a single 384-array format per chip, using the same capture reagent and similar assay conditions as those described for the Biacore experiments, except that the run buffer was supplemented with 0.5 mg/ml BSA for the interaction analysis. The LSA automates the choreography between two microfluidic modules, namely a single flow cell (SFC) and a 96 channel printhead (96PH) to deliver samples to the chip surface via a back-and-forth cycling of a fixed sample volume (200 μl in the 96PH and 250 μl in the SFC). A 384-ligand array is generated by docking the 96PH onto each of the four nested print block locations in a serial manner.

To prepare the surfaces, the SFC and 96PH were primed with run buffer (HBS-ET). The capture surface was prepared in the SFC by standard amine-coupling of goat anti-human IgG Fc to create a “lawn” onto the entire chip surface as follows. The chip was activated with a 10-min injection of a freshly prepared 1:1:1 (v/v/v) mixture of 0.4 M EDC + 0.1 M N-hydroxysulfosuccinimide (SNHS) + 0.1 M 2-(N-morpholino) ethanesulfonic acid (MES) pH 5.5. Then, goat anti-human IgG Fc was diluted to 50 μg/ml in 10 mM sodium acetate pH 4.5 and coupled for 15 min. Excess reactive esters were blocked with a 7-min injection of 1 M ethanolamine HCl pH 8.5. Final coupled levels (mean ± stdev RU across all 384 spots) were 2096 ± 79 (CMD-P), 4603 ± 334 (HC-30M), and 5731 ± 568 (CMD-200M). Anti-PD-1 mAbs were prepared at 2–5 μg/ml in run buffer and captured onto individual spots for 15 min using the 96PH. A 96-well microplate of samples consisting of duplicate sets of the mAbs, was flow printed four times to generate a 384-ligand array comprising 8 to 12 spots per mAb. The SFC was then docked over the 384-array and the run buffer was supplemented with 0.5 mg/ml BSA for the interaction analysis. Surfaces were stabilized with seven buffer analyte injections. Recombinant purified PD-1-His protein was prepared at concentrations of 0, 0.5, 1.4, 4.1, 12.3, 37, 111, 333, and 1000 nM and these samples were injected as analyte for 5 min, allowing a 20-min dissociation time. Samples were injected in ascending concentration without any regeneration in between them. Binding data from the local reference spots (interspots, representing the naked capture reagent) were subtracted from the active spots (Regions Of Interest, ROIs) and the nearest buffer blank analyte responses were subtracted to double-reference the data. The double-referenced data were fit globally to a simple 1:1 Langmuir binding model in Carterra’s Kinetic tool, allowing each spot its own *k*_a_, *k*_d_, and R_max_ value.

### Epitope binning using the Carterra LSA

Epitope binning experiments were performed in a classical sandwich assay format on an amine-coupled mAb-array as described previously,**[13]** using an HC-30M chip and regenerating with a 4:1 (v/v) mixture of Pierce^TM^ IgG Elution buffer pH 2.8 (Thermo Fisher Scientific, catalog# 21004) + 5 M NaCl. Additionally, ligand blockade was assessed using 200 nM binding sites recombinant human PD-L1 Fc chimera as analyte instead of a mAb analyte and also by premixing it at 1 μM binding sites with 50 nM PD-1.

### KinExA equilibrium experiments

The Kinetic Exclusion Assay (KinExA) experiments were conducted at room temperature (about 23°C) and in a run buffer of PBS pH 7.4 + 0.05% sodium azide + 0.01% BSA using the “fixed mAb orientation”.[33]^,13^ Thus, PD-1-His monomer was titrated (typically at final concentrations of 0.1 pM—1.2 μM as a 12-membered threefold dilution series) into a fixed concentration of an anti-PD-1 mAb (typically at a single, binding site concentration, within the range 5 pM to 50 nM) and allowed to equilibrate for up to 64 hours. These solutions were then injected over PMMA beads adsorption-coated with Fc-fused PD-1 (an asymmetric monovalent mouse-Fc-fused PD-1 construct, with total molecular weight of 70 kDa, prepared in-house by Adimab) to capture free anti-PD-1 mAb binding sites. Alexa647-labeled Goat anti-Hu IgG (H+L) (minimum cross-reactivity to bovine, horse, mouse serum proteins) was used as secondary detection. Thirteen anti-PD-1 mAbs were studied using at least two titration curves with different fixed mAb concentrations and the binding data were analyzed globally using the N-curve feature in the KinExA Pro software (version 4.3.11). The titrant was assumed to be 100% active while the apparent *K*_D_ value and the mAb’s active binding site concentration were floated (output) parameters.

### KinExA kinetic assays

The association rate constant (on-rate or *k*_a_) was determined in KinExA by using the ‘‘Kinetics, Direct” method as described before.[12] Briefly, the mAb was mixed with an antigen concentration that bound approximately 80% of the mAb in the equilibrium experiments. The starting concentrations of mAb and PD-1 were varied depending upon the mAb tested and its *K*_D_. The free mAb present in the sample was probed repeatedly, pre-equilibrium, using the same PD-1-coupled PMMA beads and Alexa647-labeled Goat anti-Hu IgG (H+L) (minimum cross-reactivity to bovine, horse, mouse serum proteins) as secondary detection. Data were analyzed utilizing the KinExA Pro software (version 4.3.11). This software graphically represents the decrease in binding signals over time and fits the collected data points to an exact solution of the kinetic differential equations for binding. From this curve, an optimal solution for the *k*_a_ is determined. The *k*_d_ is indirectly calculated from solutions for the *k*_a_ and *K*_D_.

### MSD equilibrium experiments

MSD experiments were performed as described elsewhere with the following modifications.[11, 34] Samples of 100 pM biotinylated His-tagged PD-1 were titrated with anti-PD-1 mAbs as a threefold serial dilution with top concentrations of 100, 50, or 10 nM binding sites and incubated for 18 hours at room temperature to equilibrate. Then, the samples were allowed a 2.5 min contact time with the mAb-coated and BSA-blocked MSD plates followed by secondary detection of the captured antigen with SULFO-TAG^TM^-streptavidin in 1X MSD read buffer T with surfactant. The percent free antigen was plotted as a function of titrated mAb in Prism and fit to the same quadratic equation used in the KinExA solution-based affinity measurements to provide relative *K*_D_ values for each interaction.

## Supporting information

S1 Fig LSA SPR capture kinetics on a CMD-P (flat) chip for the analog of cemiplimab (mAb23). The analog of cemiplimab (mAb23) was captured onto 12 spots at different capacities (high, medium and low), providing an overall K_D_ = 1.5 ± 0.3 nM (mean ± stdev).
(TIF)

S2 Fig Inter-assay reproducibility on the 8K and LSA instruments. (A) Results for two independent experiments performed on the 8K for 8 mAbs, where each experiment produced a single measurement per mAb. (B and C) Results for two independent 384-array based experiments on the LSA for 34 mAbs where the symbols and error bars represent the mean ± stdev for 8–12 measurements (spots) per mAb.
(TIF)

S1 Table Source of sequence information for the antibodies used in this study. Antibody ID is the name used throughout the manuscript. Reference lists the document (patent or World Health Organization-INN document) in which the antibody sequence information was disclosed (see https://www.who.int/medicines/publications/druginformation/innlists/en/ for the antibodies used in this study that are listed in the INN registry). Antibody Name is the identifier used in the associated reference.
(DOCX)

S2 Table Benchmarking the kinetics and affinities determined from the LSA (on CMD-P chip type) against those determined by KinExA (solution phase). KinExA values for *K*_D_ and *k*_a_ (with *k*_d_ deduced) are reported as the best fit (and 95% confidence interval). LSA values for *k*_a_ and *k*_d_ (with *K*_D_ deduced) are reported as the mean (and stdev) of 8–12 replicates (spots) per mAb. MAbs with very slow off-rates approaching the resolution limit of the SPR assay are reported as *k*_d_ < 4.27 x 10^−5^ (s^-1^) and are shown in bold.
(DOCX)

S1 File (XLSX)
