# Supplementary figures and images for: Assessing the binding properties of the anti-PD-1 antibody landscape using label-free biosensors
Source: PLoS One. 2020 Mar 5;15(3):e0229206. doi: 10.1371/journal.pone.0229206 (PMC7058304; doi:10.1371/journal.pone.0229206)

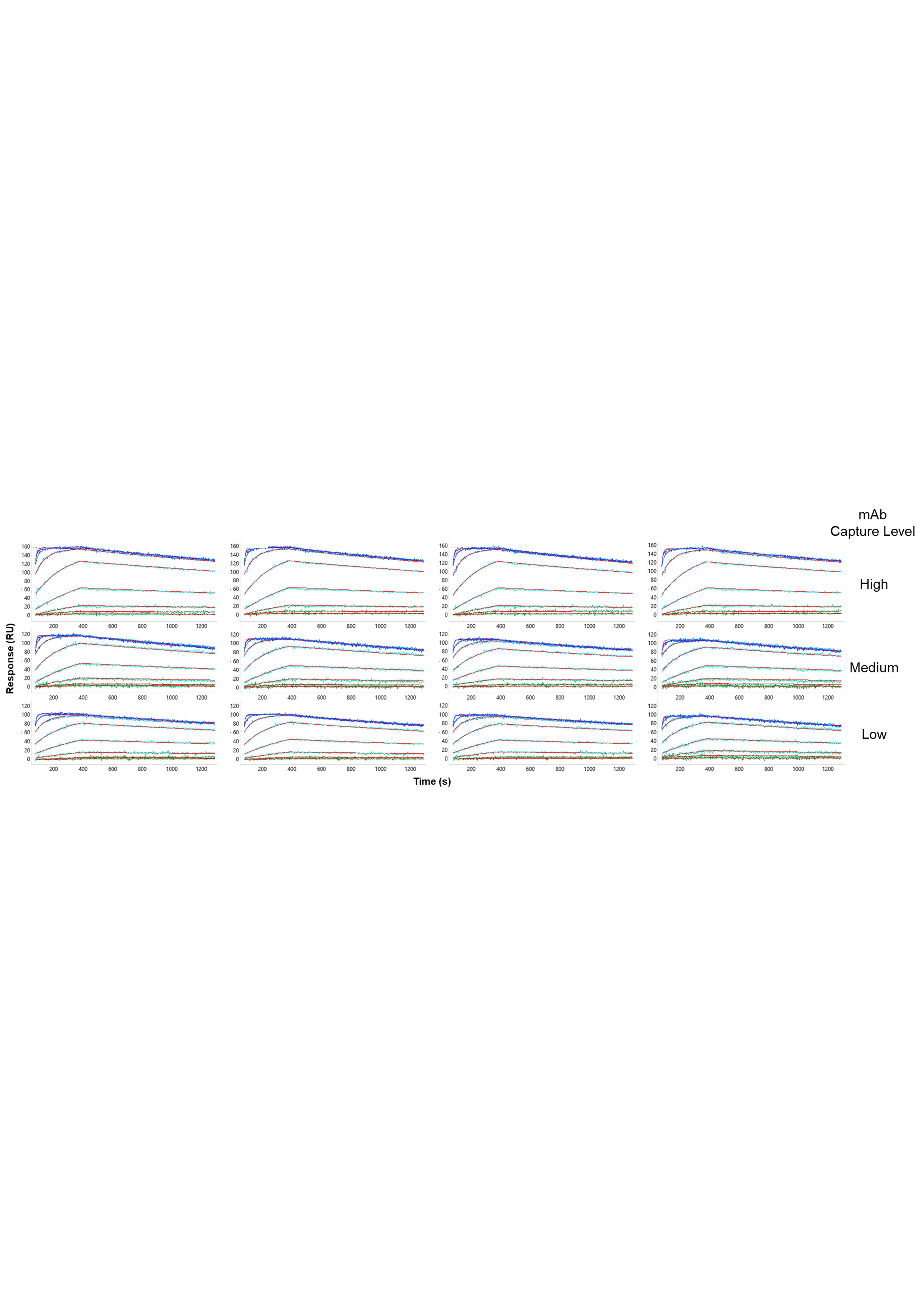

Supplement: S1 Fig — The analog of cemiplimab (mAb23) was captured onto 12 spots at different capacities (high, medium and low), providing an overall KD = 1.5 ± 0.3 nM (mean ± stdev). (TIF) [file pone.0229206.s001.TIF]

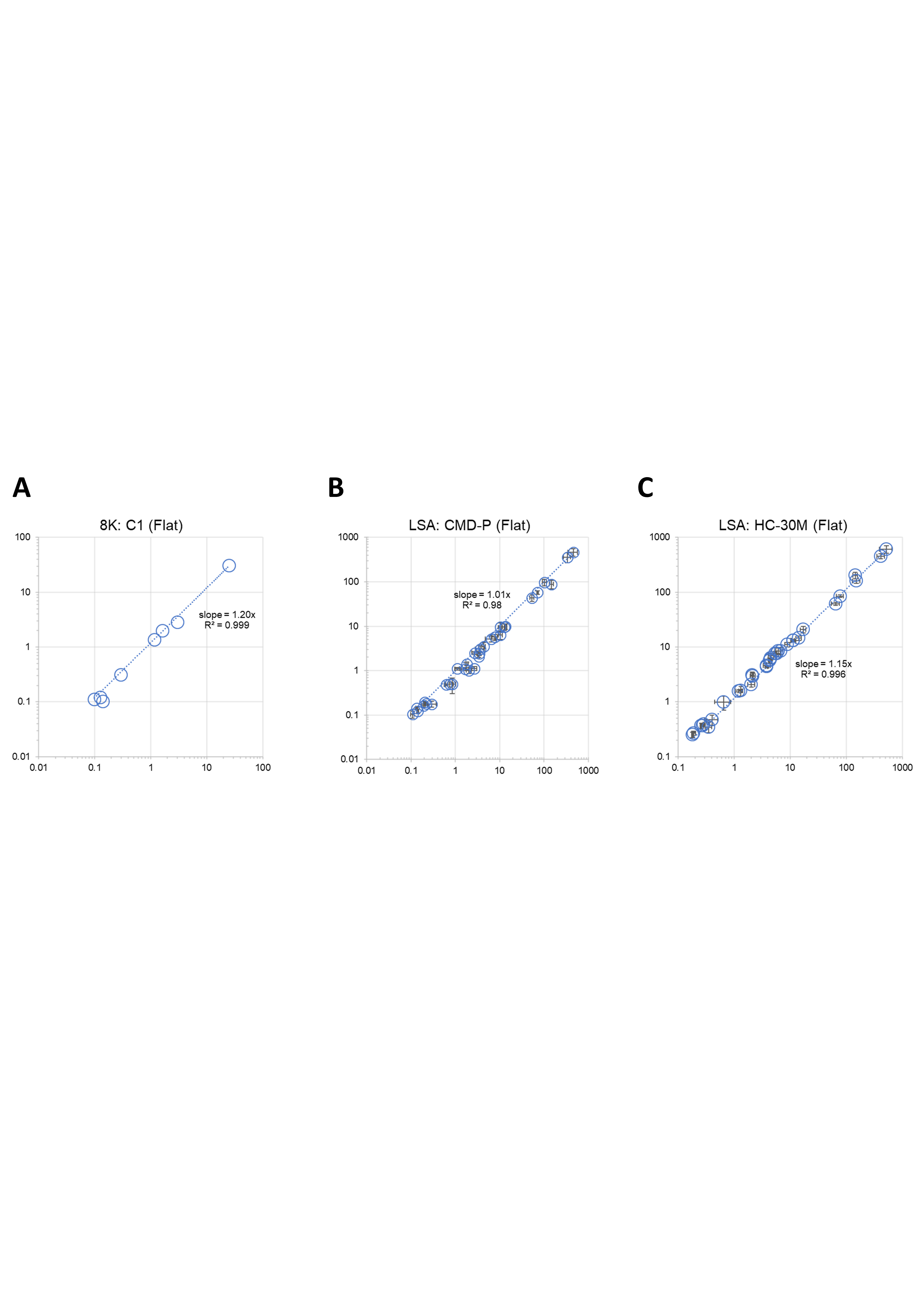

Supplement: S2 Fig — (A) Results for two independent experiments performed on the 8K for 8 mAbs, where each experiment produced a single measurement per mAb. (B and C) Results for two independent 384-array based experiments on the LSA for 34 mAbs where the symbols and error bars represent the mean ± stdev for 8–12 measurements (spots) per mAb. (TIF) [file pone.0229206.s002.TIF]
