# Supplementary material for: Assessing the binding properties of the anti-PD-1 antibody landscape using label-free biosensors
Source: PLoS One. 2020 Mar 5;15(3):e0229206. doi: 10.1371/journal.pone.0229206 (PMC7058304; doi:10.1371/journal.pone.0229206)
Supplement: S1 Table — Antibody ID is the name used throughout the manuscript. Reference lists the document (patent or World Health Organization-INN document) in which the antibody sequence information was disclosed (see https://www.who.int/medicines/publications/druginformation/innlists/en/ for the antibodies used in this study that are listed in the INN registry). Antibody Name is the identifier used in the associated reference. (DOCX) [file pone.0229206.s003.docx]

**Supplementary Table 1.** **Source of sequence information for the antibodies used in this study.** Antibody ID is the name used throughout the manuscript. Reference lists the document (patent or World Health Organization-INN document) in which the antibody sequence information was disclosed (see <https://www.who.int/medicines/publications/druginformation/innlists/en/> for the antibodies used in this study that are listed in the INN registry). Antibody Name is the identifier used in the associated reference.

| **Antibody ID** | **Antibody Name** | **Reference** |
| --- | --- | --- |
| mAb01 | 4A11 | US8008449 |
| mAb02 | h1H3Var6 | US9205148 |
| mAb03 | tislelizumab | INN |
| mAb04 | retifanlimab | INN |
| mAb05 | PD1-AB6 | US20170088618 |
| mAb06 | A1.5 | US20170044259 |
| mAb07 | 244C8-2 | US20160319019 |
| mAb08 | 388D4-3 | US20160319019 |
| mAb09 | 9B22C6C9 | WO2017016497 |
| mAb10 | 1.153.7hAb | WO2017025051 |
| mAb11 | PD1-33 | US7488802 |
| mAb12 | clone19 | US20110171215 |
| mAb13 | pembrolizumab | INN |
| mAb14 | H2M7791N | US20150203579 |
| mAb15 | balstilimab | INN |
| mAb16 | dostarlimab | INN |
| mAb17 | camrelizumab | INN |
| mAb18 | 76G5B3 | WO2017016497 |
| mAb19 | sasanlimab | INN |
| mAb20 | huPD1-0103-312 | WO2017055443 |
| mAb21 | sintilimab | INN |
| mAb22 | nivolumab | INN |
| mAb23 | cemiplimab | INN |
| mAb24 | h4B10-3 | WO2016077397 |
| mAb25 | PD1-27 | WO2016210129 |
| mAb26 | RG1H10 | US20140356363 |
| mAb27 | 1353-G08 | WO2016077397 |
| mAb28 | [12819.15384] | WO2017055547 |
| mAb29 | [12865.15377] | WO2017055547 |
| mAb30 | [13112.15380] | WO2017055547 |
| mAb31 | 1.103.11 hAbv2 | WO2017025051 |
| mAb32 | RG1H10-16C | US20140356363 |
| mAb33 | HuEH12_VH4_VK3 | US9102727 |
| mAb34 | Clone39 | US20160272708 |
| mAb35 | PD1-28 | US7488802 |
