# Supplementary material for: Assessing the binding properties of the anti-PD-1 antibody landscape using label-free biosensors
Source: PLoS One. 2020 Mar 5;15(3):e0229206. doi: 10.1371/journal.pone.0229206 (PMC7058304; doi:10.1371/journal.pone.0229206)
Supplement: S2 Table — KinExA values for KD and ka (with kd deduced) are reported as the best fit (and 95% confidence interval). LSA values for ka and kd (with KD deduced) are reported as the mean (and stdev) of 8–12 replicates (spots) per mAb. MAbs with very slow off-rates approaching the resolution limit of the SPR assay are reported as kd < 4.27 x 10^−5^ (s-1) and are shown in bold. (DOCX) [file pone.0229206.s004.docx]

**Supplementary Table 2:** **Benchmarking the kinetics and affinities determined from the LSA (on CMD-P chip type) against those determined by KinExA (solution phase).** KinExA values for *K*_D_ and *k*_a_ (with *k*_d_ deduced) are reported as the best fit (and 95% confidence interval). LSA values for *k*_a_ and *k*_d_ (with *K*_D_ deduced) are reported as the mean (and stdev) of 8-12 replicates (spots) per mAb. MAbs with very slow off-rates approaching the resolution limit of the SPR assay are reported as *k*_d_ < 4.27 x 10^-5^ (s^-1^) and are shown in bold.

| **mAb ID** | **Analog of indicated INN** | **KinExA**  ***K*_D_ (nM)** | **% active**  **mAb** | **KinExA**  ***k*_a_ (M^-1^s^-1^) x 10^5^** | **KinExA**  ***k*_d_ (s^-1^) x 10^-4^** | **LSA**  ***k*_a_ (M^-1^s^-1^) x 10^5^** | **LSA**  ***k*_d_ (s^-1^) x 10^-4^** | **LSA**  ***K*_D_ (nM)** | **LSA/KinExA ratio** | | |
| --- | --- | --- | --- | --- | --- | --- | --- | --- | --- | --- | --- |
|  |  |  |  |  |  |  |  |  | ***K*_D_** | ***k*_a_** | ***k*_d_** |
| **mAb03** | **tislelizumab** | **0.038**  **(0.03-0.047)** | **85** | **18.0**  **(16.8-19.4)** | **0.682** | **4.32 (0.374)** | **<0.427** | **0.10 (0.01)** | **2.6** | **0.2** | **0.6** |
| mAb33 |  | 1.20  (0.92-1.55) | 91 | 9.77  (9.05-10.5) | 11.7 | 3.04 (0.287) | 15.9 (1.6) | 5.23 (0.7) | 4.4 | 0.3 | 1.4 |
| mAb12 |  | 1.69  (1.22-2.31) | 80 | 3.10  (2.55-3.74) | 5.24 | 2.00 (0.149) | 18.8 (1.6) | 9.40 (1.04) | 5.6 | 0.6 | 3.6 |
| mAb05 |  | 0.55  (0.39-0.75) | 85 | 1.94  (1.68-2.21) | 1.06 | 0.56 (0.038) | 1.74 (0.29) | 3.09 (0.55) | 5.6 | 0.3 | 1.6 |
| mAb15 | balstilimab | 2.13  (1.14-3.56) | 54 | 7.63  (7.09-8.23) | 16.3 | 2.57 (0.136) | 31.6 (1.4) | 12.30 (0.86) | 5.8 | 0.3 | 1.9 |
| mAb23 | cemiplimab | 0.22  (0.17-0.28) | 99 | 5.02  (4.76-5.29) | 1.10 | 1.95 (0.276) | 2.97 (0.49) | 1.52 (0.33) | 7.0 | 0.4 | 2.7 |
| mAb27 |  | 0.35  (0.27-0.46) | 72 | 28.0  (26.6-29.6) | 9.86 | 7.74 (0.722) | 19.6 (1.9) | 2.53 (0.34) | 7.2 | 0.3 | 2.0 |
| mAb16 | dostarlimab | 0.20  (0.15-0.27) | 100 | 6.39  (5.14-8.12) | 1.30 | 2.52 (0.159) | 4.21 (0.34) | 1.67 (0.17) | 8.2 | 0.4 | 3.2 |
| mAb32 |  | 0.23  (0.17-0.29) | 118 | 34.1  (32.0-37.0) | 7.67 | 8.23 (0.687) | 16.9 (3.1) | 2.05 (0.41) | 9.1 | 0.2 | 2.2 |
| **mAb34** |  | **0.059**  **(0.045-0.075)** | **107** | **1.90**  **(1.78-2.01)** | **0.112** | **0.72 (0.056)** | **<0.427** | **0.59 (0.05)** | **10.0** | **0.4** | **3.8** |
| mAb30 |  | 0.077  (0.062-0.095) | 78 | 6.30  (6.12-6.50) | 0.485 | 1.25 (0.093) | 1.24 (0.15) | 0.99 (0.14) | 12.9 | 0.2 | 2.6 |
| **mAb09** |  | **0.14**  **(0.10-0.19)** | **68** | **0.43**  **(0.42-0.44)** | **0.0594** | **0.134 (0.008)** | **<0.427** | **3.19 (0.18)** | **23.0** | **0.3** | **7.2** |
| **mAb18** |  | **0.0035**  **(0.0024-0.0047)** | **87** | **6.40**  **(5.76-7.07)** | **0.0221** | **2.25 (0.20)** | **<0.427** | **0.19 (0.02)** | **54.8** | **0.4** | **19.3** |
